# Human Cytomegalovirus Infection Elicits Global Changes in Host Transcription by RNA Polymerases I, II, and III

**DOI:** 10.3390/v14040779

**Published:** 2022-04-09

**Authors:** Christopher B. Ball, Mrutyunjaya Parida, Ming Li, Benjamin M. Spector, Gustavo A. Suarez, Jeffery L. Meier, David H. Price

**Affiliations:** 1 Department of Biochemistry and Molecular Biology, University of Iowa, Iowa City, IA 52242, USA; christopher-ball-1@uiowa.edu (C.B.B.); mrutyunjaya-parida@uiowa.edu (M.P.); benjamin-m-spector@uiowa.edu (B.M.S.); gustavo-suarez@uiowa.edu (G.A.S.); 2 Departments of Internal Medicine and Epidemiology, University of Iowa and Iowa City Veterans Affairs Health Care System, Iowa City, IA 52242, USA; ming-li@uiowa.edu (M.L.); jeffery-meier@uiowa.edu (J.L.M.)

**Keywords:** HSV, EBV, KSHV, MHV68, PIC, transcription, productive elongation, mRNA, rRNA, tRNA

## Abstract

How human cytomegalovirus (HCMV) infection impacts the transcription of the host genome remains incompletely understood. Here, we examine the global consequences of infection of primary human foreskin fibroblasts (HFFs) on transcription by RNA polymerase I, II, and III over the course of a lytic infection using PRO-Seq. The expected rapid induction of innate immune response genes is observed with specific subsets of genes exhibiting dissimilar expression kinetics. We find minimal effects on Pol II initiation, but increased rates of the release of paused Pol II into productive elongation are detected by 24 h postinfection and pronounced at late times postinfection. Pol I transcription increases during infection and we provide evidence for a potential Pol I elongation control mechanism. Pol III transcription of tRNA genes is dramatically altered, with many induced and some repressed. All effects are partially dependent on viral genome replication, suggesting a link to viral mRNA levels and/or a viral early–late or late gene product. Changes in tRNA transcription are connected to distinct alterations in the chromatin state around tRNA genes, which were probed with high-resolution DFF-ChIP. Additionally, evidence is provided that the Pol III PIC stably contacts an upstream −1 nucleosome. Finally, we compared and contrasted our HCMV data with results from published experiments with HSV-1, EBV, KSHV, and MHV68. We report disparate effects on Pol II transcription and potentially similar effects on Pol III transcription.

## 1. Introduction

Eukaryotic nuclear transcription is primarily executed by three distinct RNA polymerases, Pol I, Pol II, and Pol III. Pol I synthesizes abundant ribosomal RNA (rRNA) from clusters of rDNA repeats, accounting for approximately 30–40% of ongoing transcription in the nucleus [1]. Pol II is the sole enzyme responsible for the synthesis of protein-coding messenger RNA (mRNA) and is highly regulated to produce the unique transcriptional profiles of diverse metazoan cell types and to execute timely responses to environmental stimuli [2,3,4]. Pol II also transcribes long noncoding RNA, microRNA, and most small nuclear RNAs (snRNA). Pol III is dedicated to the transcription of short noncoding RNAs, including all transfer RNAs (tRNA), the U6 snRNA, and the 5S rRNA, three classes of Pol III genes that differ in their promoter architecture and the involvement of general transcription factors during initiation [5,6,7]. Interactions with distinct complements of basal machinery guide these polymerases to initiate at their target core promoters. The initiation and elongation steps of transcription are regulated and rate-limiting. Pol II transcriptional regulation has been intensely investigated for decades, the efforts of which have uncovered numerous control mechanisms operating at these stages [2,3,4]. Several strategies for Pol I and Pol III transcriptional control have been reported and evidence of regulatory crosstalk between Pol I, II, and III transcription also exists [1,8,9].

Mammalian dsDNA viruses that replicate in the nucleus rely on host RNA polymerases to execute their gene expression programs. Here, most attention has been directed towards the mechanisms by which viruses co-opt host Pol II to direct the transcription of their protein-coding and long noncoding RNA genes. The human herpesviruses are dsDNA viruses that replicate largely within the host cell nucleus and purpose Pol II for the transcription of the viral genome. Decades of herpesvirus research have revealed principles of regulated Pol II initiation at viral gene promoters, which are frequently similar in structure and composition to host gene promoters, and are engaged by host and viral factors in a coordinated manner to drive a temporal cascade of viral gene expression [10,11,12,13]. More recent studies have demonstrated that paradigms of Pol II elongation control, including pausing and P-TEFb-dependent release into productive elongation [14], that are reflected at host genes also pertain to transcription on the viral genome [12,15,16,17]. Interestingly, however, several reports have shown that the viral genome differs markedly from the host genome during lytic infection in the extent to which the genome is packaged as chromatin [18,19,20,21,22]. The host genome is largely packaged in nucleosomes, restricting transcription initiation to accessible nucleosome-free regions, while the viral genome is sparsely populated by nucleosomes, a property that enables rampant transcription initiation.

Infection by herpesviruses can lead to large-scale alterations of host transcription. Alpha and gamma herpesviruses employ host shut-off mechanisms that favor productive viral gene expression. In the case of herpes simplex virus I (HSV-1), a model member of the alphaherpesvirus subfamily, the major lytic transactivator ICP4 drives a competition between the host and viral promoters for Pol II and its initiation machinery that indiscriminately diminishes transcription initiation at host promoters by late times postinfection [19]. In addition, the HSV-1 tegument protein VHS mediates the global decay of the host and viral mRNAs during early infection, a function that is essential for the full-scale production of viral progeny [23,24]. In the case of the gammaherpesviruses Kapsosi’s sarcoma-associated herpesvirus (KSHV) and murine gammaherpesvirus 68 (MHV68), a virally encoded RNA endonuclease drives widespread mRNA decay, which leads to the downmodulation of Pol II recruitment to host genes [25,26]. Here, mechanisms of anti-repression that permit robust viral gene expression are enacted. Very recent studies have shown that infection by HSV-1 and MHV68 each dramatically impact the cellular tRNA pool, actually driving an increase in the abundance of dozens of tRNAs [27,28]. Interestingly, infection was associated with a much larger increase in the abundance of immature and presumably nonfunctional pre-tRNAs than mature species, which exhibited a modest change or remained static. In one study, it was noted that the increase in pre-tRNA abundance corresponded to an increase in Pol III occupancy at tRNA genes, suggesting that Pol III transcription is induced in infected cells [28]. These studies indicated that herpesviruses may commonly regulate the tRNA pool, potentially to the benefit of productive infection, and that Pol III transcription is not subject to shut-off mechanisms akin to those that are thought to globally downregulate Pol II transcription of host genes. Notably, several other DNA viruses, including SV40 polyomavirus, Epstein–Barr virus, and adenovirus, as examples, have also been shown to increase tRNA expression, through mechanisms that involve, in part, increasing the abundance of limiting Pol III basal machinery [29,30,31,32,33]. 

Human cytomegalovirus (HCMV) is the prototypical member of the betaherpesvirus subfamily. HCMV is a nearly ubiquitous pathogen that infects more than half of the world’s population and persists lifelong in its hosts. Although infection by HCMV is typically subclinical, immunocompromised populations are at risk of unchecked HCMV replication, which can lead to systemic infection and life-threatening disease [34]. Moreover, intrauterine HCMV infection is a common cause of morbidity in newborns and is the leading infectious cause of birth defects in the United States [35]. Dissimilar to HSV-1, KSHV, and MHV68, HCMV is not thought to elicit global host shutoff through transcriptional or post-transcriptional mechanisms. HCMV infection does induce innate and adaptive immune responses and profoundly impacts the levels of hundreds of mRNAs encoding factors that are involved in cell cycle progression, DNA replication, formation of the extracellular matrix, vesicular trafficking, and metabolism [36,37,38]. The effects of HCMV infection of Pol I and Pol III transcription have not, to our knowledge, been directly examined. Here, we further explore the transcriptional and epigenetic changes driven by lytic HCMV infection at host genes using PRO-Seq, a method that globally profiles Pol I, Pol II, and Pol III nascent transcripts [39,40] and DFF-ChIP, which enables complexes such as the Pol I, II, and III preinitiation complexes, in addition to their local chromatin environment, to be probed with high resolution [18].

## 2. Materials and Methods

### 2.1. Viruses, Cells, and Conditions of Infection

Primary human foreskin fibroblasts (HFFs) were derived from deidentified, discarded newborn infant foreskins. HFFs were maintained in Minimum Essential Medium (Gibco, 11095080) that was supplemented with 5% fetal bovine serum (Gibco, Waltham, MA, USA, 26140079) and 1% penicillin–streptomycin (Gibco, Waltham, MA, USA, 15140122). All infections were performed with contact-inhibited HFF and TB40/E BAC4 virus at an MOI of 3 as previously described [41]. Cells were treated with 1 µM Flavopiridol (Flavo) or DMSO vehicle control during the last hour of infection, and phosphonoformic acid (PFA, Sigma Aldrich, St. Louis, MO, USA) was added to the media at 400 µg/mL for the indicated conditions as described previously [41].

### 2.2. PRO-Seq and PRO-Cap

Spike-in quantitative PRO-Seq data analyzed in this study from TB40/E infected primary HFF were previously published and were prepared exactly as described in [18]. PRO-Cap data from HFF infected with TB40/E for 72 h were utilized to define transcription start sites at Pol II genes for truQuant analysis of pause region and gene body counts. The PRO-Cap dataset was previously published and prepared as described in [18]. All data are available at NCBI GEO GSE185763.

### 2.3. DFF-ChIP

DFF-ChIP data analyzed in this study from TB40/E infected primary HFFs were previously published. Sample processing and library construction were carried out exactly as described by Spector et al. [18]. Data are available at NCBI GEO GSE185763.

### 2.4. Generation of Tracks

Stranded PRO-Seq data tracks viewable with the UCSC genome browser (human genome assembly hg38) were generated as previously described [41], in a process that first involved trimming of adapter sequences with trim_galore v0.6.0 (available online: https://github.com/FelixKrueger/TrimGalore/releases/tag/0.6.6 (accessed on 3 March 2019)), strand-specific alignment of paired-end sequences with bowtie v1.2.3 (available online: http://bowtie-bio.sourceforge.net/index.shtml (accessed on 5 July 2019)), UMI-based removal of PCR duplicates with dedup (available online: https://github.com/P-TEFb/dedup (accessed on 14 February 2019)), and subsequent conversion of the deduplicated, aligned data into bedGraphs and bigwigs. Read coverage at each position was normalized by multiplication of spike-in correction factors. Spike-in correction factors were computed based on total reads in the library and reads mapping to the spike-in genome, as previously described [42], all details of which are provided in Supplementary Data File, PRO-Seq Stats. Tracks for DFF-ChIP data were generated as previously described.

### 2.5. TruQuant Analysis, Gene Body Clustering, FragMaps, and Metaplots

The pause region and gene body intervals of 11,593 genes transcribed in the human genome after 72 hpi HCMV infection were generated from the HFF PRO-Cap dataset using the truQuant program with default settings (available online: https://github.com/meierjl/truQuant (accessed on 31 August 2020)). The bedtools coverage program v2.27.1 [43] (available online: https://bedtools.readthedocs.io/en/latest/index.html (accessed on 14 December 2017)) was used to determine the total number of 5′ or 3′ ends in pause region or gene body intervals, respectively. The gene body counts for all infected time points were divided by the count from the uninfected data to calculate the fold change. Genes with their associated fold changes at various infected time points were hierarchically clustered using the pheatmap library in R. Pearson correlation was chosen as the clustering distance metric and the number of clusters was retained at 10. The number of clusters was chosen using the within-cluster sum of squares (wcss) method in Python’s sklearn.cluster package and KMeans library. All genes and their associated fold change profiles were scaled independently using the z-score formula and colored using the pheatmap function in R. Average gene body changes for all clusters were calculated using a Python script by averaging fold changes across time points and plotted as line graphs using MS Excel. Gene sets for specific clusters were plotted independently using the pheatmap function in R.

Median fold changes of 11,593 genes across all infected time points were determined using a Python script from the list of fold changes calculated by first dividing the total number of 5′ end counts in their pause regions and the total number of 3′ end counts in their gene body regions with associated counts of pause region and gene body regions from the uninfected datasets. Median fold changes were plotted using MS Excel. The pause ratio was computed for each gene in the DMSO-treated samples by dividing the number of 5′ ends in the pause region by the pause region length (150 bp), and then dividing this number by ratio of the number of 3′ ends in the gene body over the gene body length for each gene (based downstream of the pause region to the annotated CPS). Pause ratios for all genes in each sample were plotted as boxplot in MS excel.

The sum of H3K4me3 read densities across genomic intervals between −500 and +3000 bp from the MaxTSS of all genes in clusters 1, 3, and the repressed set were generated using bedtools coverage program. Read densities were plotted using MS Excel. FragMaps were generated from the HFF uninfected and 48 hpi H3K4me3 fragments of size between 18 bp and 400 bp that were present in the genomic intervals between −/+500 bp from the MaxTSSs of genes in the repressed set using the fragMap program (available online: https://github.com/P-TEFb/fragMap (accessed on 13 August 2021)).

### 2.6. Bioinformatics Analysis of tRNA Transcription

An annotation of 429 tRNA genes was downloaded from the genomic tRNA database (available online: http://gtrnadb.ucsc.edu/ (accessed on 1 December 2021)). The mature 5′ ends of tRNAs were used for further analysis. In total, 235 tRNA genes out of 429 were used to compare tRNA transcription between uninfected and infected time points. These genes exhibited most initiation from the position upstream of their mature 5′ end. Strand specific transcription of tRNA genes was measured at their 1 bp upstream position using bedtools coverage program [43]. Scatter plots of uninfected HFF PRO-Seq data were plotted against the 4 hpi, 12 hpi, 24 hpi, 48 hpi, 72 hpi, and 72 hpi PFA-treated samples using MS Excel. Read coverage of the 235 tRNA genes at the position 1 bp downstream from their mature 3′ ends was calculated using bedtools coverage program. The program was used to determine the total number of strand-specific fragments at this position for 48 hpi HCMV infection and uninfected DMSO-treated datasets. Fold change calculation was performed by dividing the fragment counts of 48 hpi HCMV infection over uninfected DMSO-treated datasets. The scatter plot of the fold change was plotted using MS Excel.

Heatmaps of −50 bp and +200 bp genomic intervals centered on the mature 5′ ends of 235 tRNA genes were created using the heatmap program (available online: https://github.com/P-TEFb/Heatmap (accessed on 4 March 2022)). The 5′ and 3′ ends of HFF PRO-Seq uninfected and 48 hpi DMSO-treated datasets were generated following the steps mentioned in our metaplots tutorial (available online: https://github.com/P-TEFb/Metaplot-and-Counting-fragments (accessed on 3 March 2022)). Parameters for these heatmaps were strand = yes, order = DESC, avgrows = 1, width = 2, height = 4, black = user-specified (1/10th of the maximum value in the heatmap table), and gamma = 0.5.

### 2.7. Motif Enrichment Analysis

Motif enrichment was performed using a previously documented motif analysis software [44] (available online: https://regulatory-genomics.org/motif-analysis/method/ (accessed on 17 February 2022)). Input regions were bed files containing 400 bp promoter regions padded around the 150 bp pause region (−200 bp upstream, +50 bp downstream) for genes in clusters 1, 3, 4, and 8. The background file consisted of such promoter regions from all 11,594 active gene promoters. Transcription factor motifs enriched in experimental regions versus the background were determined using the rgt-motifanalysis matching function with script --input-files input/experimental.bed input/Background.bed --organism hg38 --remove-strand-duplicates. Next, the statistical significance of enrichment over background was measured using the rgt-motifanalysis enrichment function, which performed a Fisher’s exact test for each enriched transcription factor binding motif.

### 2.8. Statistics

Statistics for motif enrichment analysis were reported by the motif analysis software. For correlations of tRNA read coverage between DMSO and Flavo PRO-Seq datasets, a Pearson’s correlation coefficient was computed in Microsoft Excel. 

## 3. Results

### 3.1. Global Changes in Pol II Transcription during HCMV Infection

**Figure 1:**
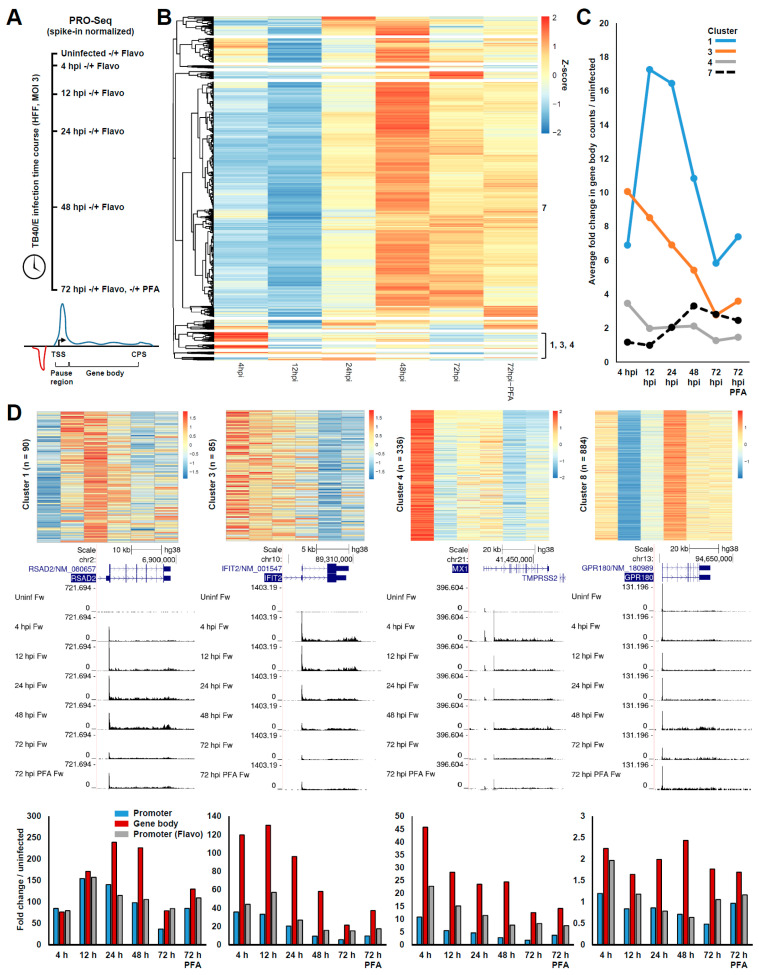
Effects of HCMV infection on Pol II transcription. (**A**) Schematic depicting experimental design. Spike-in quantitative PRO-Seq was performed with samples derived from contact-inhibited HFFs that were mock-infected or infected for 4, 12, 24, 48, or 72 h. For all time points, an additional sample in which the cells were treated with Flavo for the final hour of infection to block Pol II pause-release was collected. For the 72 h time point, the effect of blocking HCMV replication by treatment with PFA from the onset of infection was also tested −/+ Flavo during the final hour of infection. At the bottom, a diagram depicting the pause and gene body regions quantified for analysis is shown; (**B**) Heatmap displaying hierarchical clustering of ratios in PRO-Seq gene body counts, which were computed for each gene at each time point over the uninfected control and are represented as Z-scores, which were computed for each gene. Certain clusters are identified at the right of the heatmap; (**C**) plot of average fold change in gene body counts for all genes within clusters 1, 3, 4, and 7 along the infection time course; (**D**) top: blown up views of heatmaps representing genes in clusters 1, 3, 4, and 8. Legends represent the Z-score. Middle: UCSC Genome Browser snapshots showing forward strand (Fw) PRO-Seq data from DMSO-treated samples, for example, genes representing the above clusters. Bottom: graphs displaying fold changes in pause region and gene body counts from DMSO-treated samples and fold change in pause region counts from Flavo-treated samples along the infection time course for the above genes.

To investigate the effects of HCMV infection on transcription of the host genome, we carried out a time course of HCMV infection (TB40/E strain, MOI 3) in contact-inhibited HFF and performed spike-in quantitative PRO-Seq. Libraries were prepared from uninfected cells and cells infected for 4, 12, 24, 48, and 72 hpi, which sample the early-stage of lytic infection (4, 12 hpi), a mid-stage of infection close to the onset of viral genome replication (24 hpi), and late-stage (48, 72 hpi) that follows the onset of genome replication. Additional samples in which the uninfected or infected cells were treated for the final hour prior to harvest with Flavopiridol, a P-TEFb inhibitor that blocks Pol II pause-release [45], were prepared. In addition, a sample in which cells were infected for 72 h and treated from the onset of infection with phosphonoformic acid (PFA), an HCMV replication inhibitor, was prepared, and this condition was additionally combined with the Flavo treatment during the final hour of infection (Figure 1A). PRO-Seq involves the rapid isolation of native nuclei followed by a nuclear-run on reaction in the presence of biotinylated nucleotides [39,40]. Total RNA was afterwards isolated and biotinylated nascent transcripts were enriched with streptavidin beads under stringent conditions for the subsequent library construction. The efficiency of adding consecutive biotinylated nucleotides to the nascent RNA chain was extremely low, at least for Pol II [39]; this affords single-nucleotide resolution not offered by other run-on and metabolic labeling approaches. PRO-Seq does not distinguish between transcription by Pol I, II, or III, such that a single experiment theoretically enables one to query transcription by any of these polymerases.

The total number of sequenced reads in each sample, numbers of reads mapping to the host, viral, and spike-in (Spodoptera) genomes, percentage of reads mapping to the viral genome, and spike-in normalization factors for each sample are shown in Supplementary Data File, PRO-Seq Stats. All subsequent analyses utilized spike-in normalized data. Initial focus was directed towards the effects of HCMV infection on Pol II transcription. PRO-Cap data for HFFs infected for 72 h with TB40/E were utilized to annotate a set of transcriptionally active genes (n = 11,594) with our previously described tsrFinder and truQuant algorithms [12,41]. For every active gene, the number of 5′ ends in the Pol II pause region, defined by the truQuant algorithm [41], and the number of 3′ ends in the gene body region, beginning at the base after the pause region end and extending to the annotated cleavage and polyadenylation site (CPS), were quantified for each PRO-Seq dataset (Figure 1A, Supplementary Data File). As gene body counts were more highly correlated with transcriptional output than pause region counts, we took the ratio of gene body counts for all genes at every time point over the uninfected control and utilized these ratios to perform hierarchical clustering and identify groups of genes that exhibited similar patterns of change along the infection time course (Figure 1B). Ten clusters were selected using the elbow method. Clusters five, six, and seven, containing a total of 9605 (82%) active genes, reflected a major, unexpected trend in the data, which was a gradual increase in gene body counts along the time course, peaking at 48 or 72 h. Genes within clusters one (n = 90), three (n = 85), and four (n = 336) exhibited a sharp increase in gene body counts at 4 hpi over the uninfected control, but differed with respect to change in gene body counts at later time points (Figure 1C,D). These clusters were, as expected, highly enriched for genes involved in innate immunity, as revealed by the pathway and ontology analysis performed with Enrichr [46] (Supplementary Data File, GO-Terms and BioPlanet Pathways). For example, the cluster one gene RSAD2, an interferon-stimulated gene (ISG) [47], exhibited a great induction at 4 hpi that further increased at 12 hpi and peaked at 24–48 hpi (Figure 1D). The cluster three gene IFIT2, an ISG [48], was greatly induced at 4 hpi and gradually decreased as infection progressed. The well-characterized ISG MX1 [48] fell within cluster four, and was sharply induced at 4 hpi and exhibited a reduced but relatively stable level of gene body transcription between 12 and 48 hpi. Cluster eight genes (n = 884) contained genes such as the putative interferon-responsive gene GPR180 [49], which was modestly induced at 4 hpi, decreased at 12 hpi, and then in association with most other active genes, exhibited an upward trend in transcription during the later stages of infection (Figure 1D). 

Intriguingly, we found that the trends in the pause region and gene body counts, plotted as a fold change over the uninfected control for each example gene (Figure 1D, lower panels), were frequently inconsistent, which indicated an uncoupling between initiation and release into productive elongation. Following initiation, paused Pol II either terminates or undergoes a P-TEFb-dependent release into productive elongation, a step that is known to be regulated and govern transcriptional output [50]. The amount of paused Pol II observed in the presence of Flavo is in part dependent on termination. However, due to the inhibition of P-TEFb, this amount is not influenced by release into productive elongation, and, therefore, more accurately represents the levels of initiation. Accordingly, treatment with Flavo partially corrected discrepant trends in the pause region and gene body counts in the presence of DMSO along the course of infection, indicating that a substantial fraction of paused Pol II at these genes entered into productive elongation (Figure 1D).

These patterns were representative of dozens of other genes induced by infection, many of which are known ISGs. The transcriptional mechanisms that control the induction of ISGs have been intensely investigated, but remain incompletely understood and are complex, involving numerous transcription factors (TFs) operating concertedly and dynamically across time [51,52,53]. This transcriptional response is wired to be transient, at least in part to limit the deleterious effects of long-term proinflammatory gene expression. In addition, HCMV encodes a large arsenal of immunomodulatory factors that counter innate and adaptive immune responses. HCMV is known to subvert innate immune responses through transcriptional [54], post-transcriptional [55], and post-translational mechanisms [56]. Our PRO-Seq data, which highlighted the dynamic nature of ISG induction, measured nascent transcription, and were not significantly impacted by post-transcriptional mechanisms regulating RNA abundance, represent a minable source of information that may be useful in unraveling the direct transcriptional response to HCMV infection and innate immune signaling more broadly. In this regard, we investigated the promoter regions of genes in clusters one, three, four, and eight for enriched TF motifs using a motif analysis software [44] (Figure S1). The region queried was 400 bp, padded around the pause region, which captured most of the sequence contained within a typical nucleosome-free region (NFR), and enrichment was tested relative to a background consisting of equivalent regions from all 11,594 active host gene promoters. Interestingly, genes in clusters three and four, which were enriched for ISGs that were most induced shortly after infection at 4 hpi, exhibited a highly significant enrichment of IRF and STAT-family TF motifs in the proximal promoter region. Cluster one genes, which exhibited a delayed but a robust induction, differed in that their proximal promoters were only weakly enriched for IRF-family TF motifs, and no enrichment of STAT-family TF motifs was detected. By contrast, cluster eight genes, which were mildly induced at 4 hpi, exhibited a weak enrichment for STAT and IRF-family TF motifs.

### 3.2. Late HCMV Infection Is Associated with Increased Rates of Release into Productive Elongation at Host Pol II Genes

**Figure 2:**
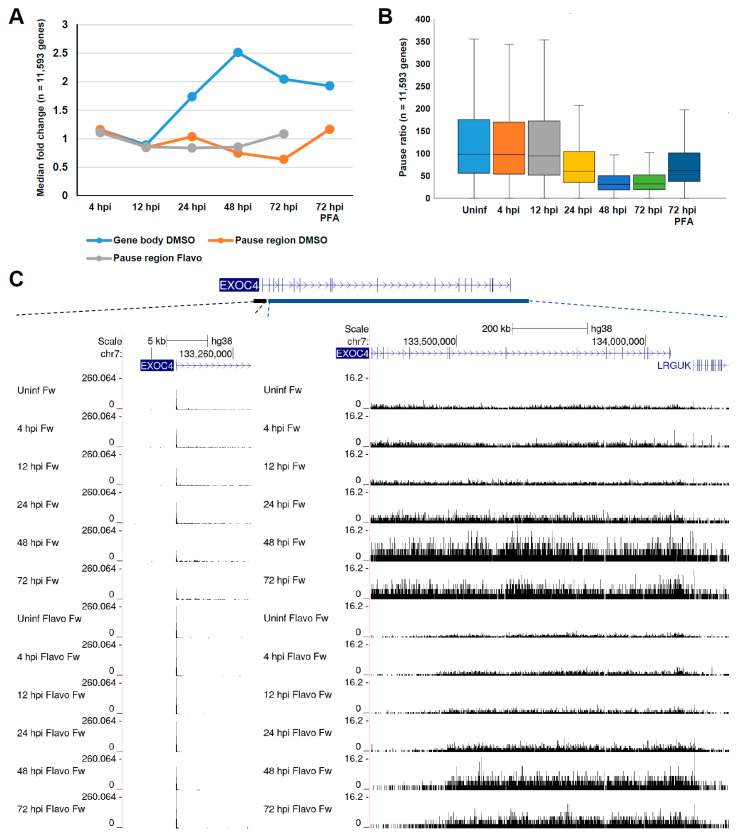
Late HCMV infection is associated with increased rates of release into productive elongation. (**A**) Graph displaying median fold change in pause region and gene body counts from DMSO-treated samples and fold change in pause region counts from Flavo-treated samples along the infection time course; (**B**) boxplot displaying calculated pause ratios for each gene across all samples; (**C**) genome browser snapshots of the pause region and gene body of the EXOC4 gene, showing a modest decrease in pausing and a substantial increase in amounts of productively elongating Pol II at late times postinfection.

The observation that gene body transcription trended upwards during late HCMV infection was unexpected and further explored. The median fold change in the pause region and gene body counts along the infection time course, as well as the median fold change in the number of pause region counts in the presence of Flavo which blocked the release into productive elongation, was plotted (Figure 2A). Clearly, the median number of gene body counts increased substantially along the course of infection, while the number of pause region counts modestly decreased. Blocking the release into productive elongation led to an increase in counts in the pause region at late times postinfection. Extending this analysis, the pause ratio for each gene, which measures the proportion of Pol II engaged in pause regions versus gene bodies (after normalization to the size of the regions), was calculated (Figure 2B). In uninfected cells and cells infected for 4 or 12 hpi, most genes exhibited a high pause ratio, but a near-global downward shift was detected at 24 hpi and the pause ratio dramatically reduced at 48 and 72 hpi, reflecting the widespread increase in gene body counts. Interestingly, blocking HCMV replication with PFA in cells infected for 72 h resulted in increased pause ratios compared to the 72 h time point, mirroring the result acquired at 24 hpi (pre-replication), and suggesting that the substantial decrease in pause ratios observed at late times may have required viral genome replication and/or increasing levels of viral transcription. The above described trends were well represented by the PRO-Seq signal over the EXOC4 gene, which, for clarity, was broken into the pause and gene body regions (Figure 2C). This result was also captured in cells treated with Flavo, as there was a notable increase in the ‘receding wave’ of Pol II elongating down the long EXOC4 gene at late times postinfection, following the acute inhibition of pause release.

### 3.3. Chromatin Changes at Pol II Genes Activated and Repressed by HCMV Infection

**Figure 3:**
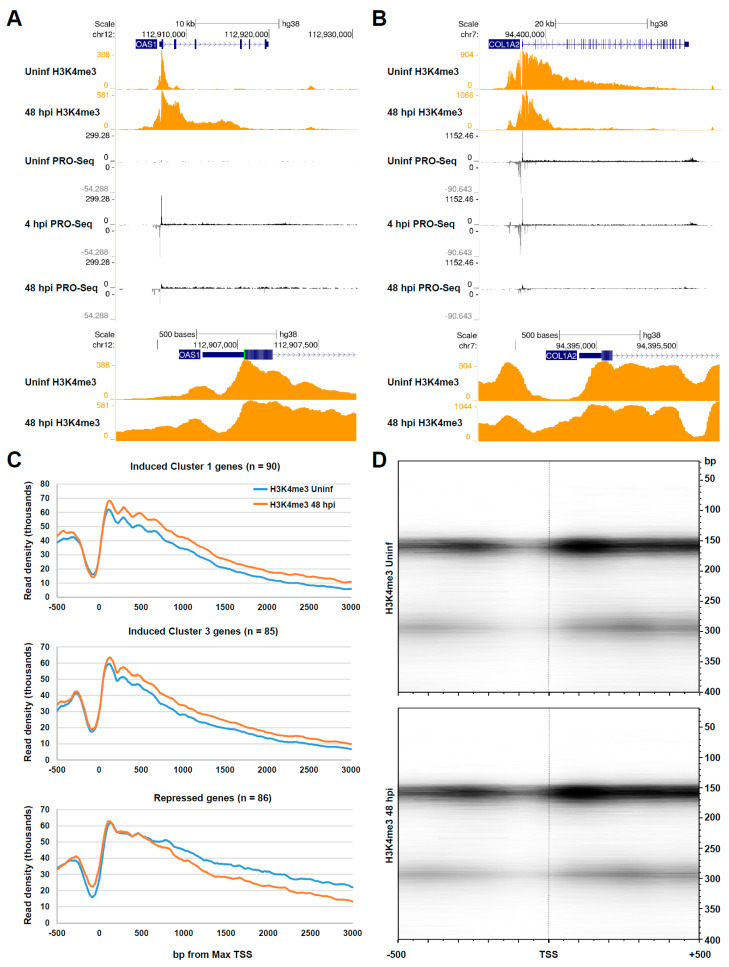
Changes in the chromatin status of genes induced and repressed by HCMV infection. (**A**) Top: genome browser snapshot of PRO-Seq data for the induced OAS1 gene in uninfected HFFs and HFFs infected for 4 or 48 h, and H3K4me3 DFF-ChIP data in uninfected HFFs and HFFs infected for 48 h. Bottom: blown up view of H3K4me3 DFF-ChIP data at the OAS1 promoter region; (**B**) top: Genome browser snapshot of PRO-Seq data for the repressed COL1A2 gene in uninfected HFFs and HFFs infected for 4 or 48 h, and H3K4me3 DFF-ChIP data in uninfected HFFs and HFFs infected for 48 h. Bottom: blown up view of H3K4me3 DFF-ChIP data at the COL1A2 promoter region; (**C**) metaplots of H3K4me3 DFF-ChIP data in uninfected HFFs and HFFs infected for 48 h for cluster 1 genes and cluster 3 genes, which were enriched for induced ISGs and repressed genes. Summed H3K4me3 signals were plotted over a region spanning −500 to +3000 relative to the Max TSS; (**D**) FragMaps of H3K4me3 DFF-ChIP data at repressed genes in uninfected HFF and HFF infected for 48 h.

Next, we sought to address whether HCMV infection drove changes in the chromatin state of differentially transcribed genes. We recently reported DFF-ChIP for H3K4me3, an epigenetic modification that was enriched on the positioned nucleosomes flanking active Pol II promoters, in uninfected HFFs and HFFs infected for 48 h with HCMV TB40/E. DFF-ChIP utilizes the dsDNA-specific endonuclease DFF to digest chromatin prior to IP [18]. Importantly, DFF exhibits very little propensity to digest within nucleosomes, a property that enables sites of H3K4me3 nucleosome occupancy to be defined with high resolution [57]. ISGs are transcribed at very low levels under normal conditions, but their promoters are, nevertheless, reported to be open and primed for induction [53,58]. Congruent with these descriptions, we observed that the ISG OAS1 contained a well-defined nucleosome-free region (NFR) in uninfected cells flanked by H3K4me3-modified nucleosomes (Figure 3A). At 48 hpi, the NFR was unaltered, but the profile of H3K4me3 extended deeply into the gene body, which was consistent with the current model that Pol II elongation is associated with H3K4me3 installment [59,60]. H3K4me3 was recognized by the TAF3 subunit of TFIID [61] and the chromatin remodeler CHD1 [62], which is thought to also tether components of spliceosome. Thus, the increased load of H3K4me3 may function to facilitate the robust initiation and elongation at ISGs. 

By contrast, 86 genes were observed to undergo a significant repression of gene body transcription across consecutive time points of the infection time course. For example, the transcription of the normally active COL1A2 gene was repressed more than five-fold at 48 hpi compared to the uninfected control (Figure 3B). Repression was initiated rapidly after infection, at 4 hpi, and deepened over time. H3K4me3 DFF-ChIP revealed a broad profile of the modification that extended far into the gene body in uninfected cells and was considerably retracted at 48 hpi. Strikingly, the COL1A2 NFR exhibited an encroachment of H3K4me3 nucleosomes at 48 hpi compared to the uninfected control, likely a consequence of the reduction in promoter activity. A gene ontology and pathway analysis revealed that this set of repressed genes was enriched for proteins involved in the organization of the extracellular matrix, including several collagens, lumican, and metallopeptidases (Supplementary Data File, GO-Terms, BioPlanet Pathways). The HCMV-induced repression of these genes has been reported [36,37,63], and may be linked to the well-documented observation that HCMV-infected cells exhibit cytopathic cell rounding and compromised substrate adhesion [64,65]. Our data added that the repression of these genes initiated rapidly following infection through a mechanism that ultimately resulted in the closure of the promoter NFRs. Metaplots were generated for H3K4me3 in uninfected cells and cells infected for 48 at all cluster one and cluster three genes, which were enriched for induced ISGs, in addition to the 86 defined repressed genes. These metaplots revealed an overall unchanged NFR for cluster one and three genes and increased H3K4me3 levels downstream in gene bodies. Repressed genes exhibited a retraction of the H3K4me3 signal from gene bodies and an increase in H3K4me3 nucleosomes within the NFR (Figure 3C). To view these data another way, we generated fragMaps of the H3K4me3 signal at repressed genes in uninfected cells and cells infected with HCMV for 48 h. A fragMap is a quantitative 2D plot of the level of fragments of various sizes versus the position [18], here centered on the MaxTSS for every gene represented. The fragMaps revealed a well-positioned +1 nucleosome downstream of the TSS, and in infected cells, fragments corresponding to a nucleosome in size (~150 bp) further extended into the NFR, which was consistent with promoter occlusion (Figure 3D).

### 3.4. HCMV Infection Alters Transcription of the 45S rDNA Repeat by Pol I

**Figure 4:**
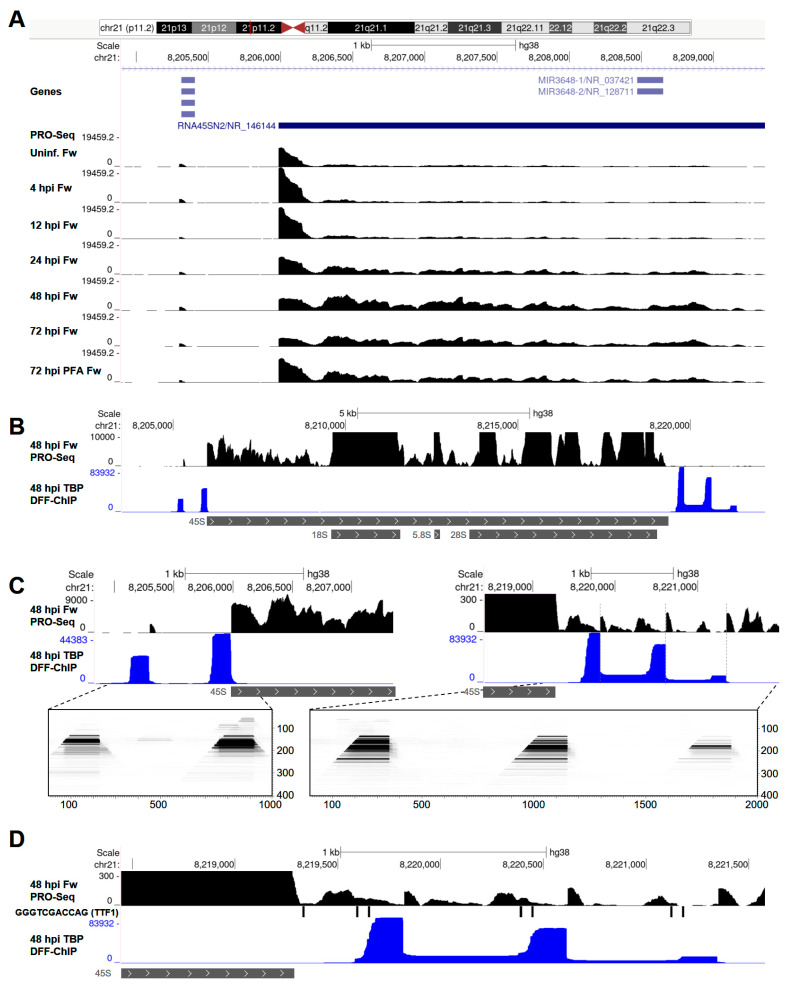
HCMV infection impacts Pol I transcription of rDNA. (**A**) UCSC Genome Browser snapshot of PRO-Seq data over the 45S promoter region and 5′ portion of 45S not retained in mature rRNA; (**B**) genome browser snapshot of TBP DFF-ChIP data at an rDNA repeat, with a schematic depiction of 45S, 18S, 5.8S, and 28S below; (**C**) left: blown up view of TBP DFF-ChIP data at the 45S promoter region, indicating the positions of Pol I PICs, and a fragMap below corresponding the region indicated by the dashed lines. Right: blown up view of TBP DFF-ChIP data at the 45S downstream region, indicating the positions of Pol I PICs, and a fragMap below corresponding the region indicated by the dashed lines; (**D**) UCSC Genome Browser of TBP DFF-ChIP and PRO-Seq data at the 45S downstream region documenting evidence that the downstream PICs drive transcription initiation, and that the PICs are intimately associated at their upstream end with TTF1 termination sites.

Ribosomal RNA is synthesized by Pol I, which transcribes a single 45S precursor RNA that is, subsequently, processed to yield the 18S, 5.8S, and 28S ribosomal RNAs; 45S is encoded within an rDNA repeat that exists as a tandem array found on the short arms of chromosomes 13, 14, 15, 21, and 22. In the present hg38 assembly, eight rDNA repeats are annotated. Due to the remarkable abundance of ribosomal RNA in cells, mature 18S, 5.8S, and 28S rRNAs can contaminate PRO-Seq libraries despite extensive selection for biotinylated nascent transcripts, but we found that thorough washing eliminated most of this contaminating background signal over mature rRNA. To eliminate all background, the analysis of Pol I transcription was restricted to the 45S promoter region and 5′ portion of 45S that is not retained as a mature transcript. Initiation by Pol I occurred at a single sharp start site. Pol I is not thought to undergo promoter-proximal pausing like Pol II. However, unexpectedly, we observed an accumulation of transcripts all starting at the 5′ end of the 45S gene, extending up to about 200 bp downstream. This was reminiscent of Pol II pausing, though the transcripts associated with Pol I were about 2–3 times longer than typically seen for paused Pol II (Figure 4A). Very interestingly, as infection progressed, this signal corresponding to ‘paused Pol I’ was diminished, and a more elongating Pol I was detected downstream. At 72 hpi, this effect on Pol I transcription was partially reversed by treatment with PFA, suggesting that the apparent stimulation of Pol I elongation may depend on viral genome replication, increasing levels of viral mRNA and/or the involvement of a viral factor. These effects were also observed in the presence of Flavo, indicating that P-TEFb is not involved in regulating Pol I transcription and suggesting that the differences in Pol I transcription during late infection were not directly coupled to ongoing Pol II productive elongation (Figure S2). 

We recently reported DFF-ChIP for TATA-binding protein (TBP) [18], a core general transcription factor that assembles into Pol I, Pol II, and Pol III preinitiation complexes (PICs), in HFFs infected for 48 h with HCMV TB40/E. Our DFF-ChIP approach was demonstrated to define the boundaries of Pol II PICs with an unprecedented resolution. Although not reported in the original publication, major TBP features were detected in the rDNA loci. Two were in the 45S promoter region and unexpectedly three other TBP-containing complexes were detected downstream of the region encoding the 45S precursor rRNA (Figure 4B,C). The two upstream features were present at all eight annotated rDNA repeats; they corresponded to PICs formed over the previously described spacer promoter and the rRNA promoter [66] (Figure 4C, left). Downstream of the 45S gene, in the five rDNA repeats that contained sequence information, three apparent PICs spaced approximately 700 bp apart were detected (Figure 4C, right). All of the PICs (both the upstream and downstream) drove transcription initiation, albeit at vastly different levels, starting 5–10 bp beyond the downstream edge of the PIC. Only initiation from the main PIC located immediately upstream of the 45S gene drove 45S transcription. Transcription from the downstream PICs was slightly increased during late HCMV infection, while transcription from the upstream spacer promoter was not impacted either at the level of initiation or elongation. A set of TBP fragMaps for the example shown demonstrated that these PICs all had similar 160 bp footprints (Figure 4C). Presumably, all of the PICs were occupied by the TBP-containing SL1 complex downstream of a region bound by UBF [67]. DFF digestion between these two subcomplexes was detected in the main PIC driving rRNA transcription (Figure 4C, left). The rRNA promoter was also unique among the regions directing PIC assembly, as it alone contained a TATAT sequence upstream of the TSS. It is not clear what role the downstream PICs possess, but it was noted that their upstream edges were intimately associated with TTF1 termination sites (Sal boxes, GGGTCGACCAG) [68] and, thus, could be involved in preventing transcription read-through into adjacent rDNA repeats (Figure 4D). 

### 3.5. HCMV Infection Dramatically Impacts tRNA Transcription by Pol III

In light of recent reports suggesting that infection by both alpha- and gammaherpesviruses induces tRNA expression [27,28,31], we next investigated whether HCMV, a betaherpesvirus, also affects tRNA transcription. The measurement of tRNAs by sequencing approaches has been limited, as mature tRNAs are refractory to reverse transcription due to their extensive base modifications and secondary structure. Several recently developed approaches have enabled better quantitative profiling of tRNAs [69,70,71,72]. As tRNAs are thought to be post-transcriptionally processed and modified, the measurement of nascent Pol III transcripts theoretically eliminates the restriction to sequencing posed by base modifications. In addition, secondary structure may be minimal on tRNAs that are not completely transcribed. Finally, mature tRNAs are extremely stable, with a half-life of days [72], and, thus, small changes measured in the mature tRNA population may belie major changes at the level of transcription. PRO-Seq could, therefore, be uniquely informative of changes in tRNA expression, with the caveat that post-transcriptional mechanisms also contribute to the abundance of functional tRNAs.

**Figure 5:**
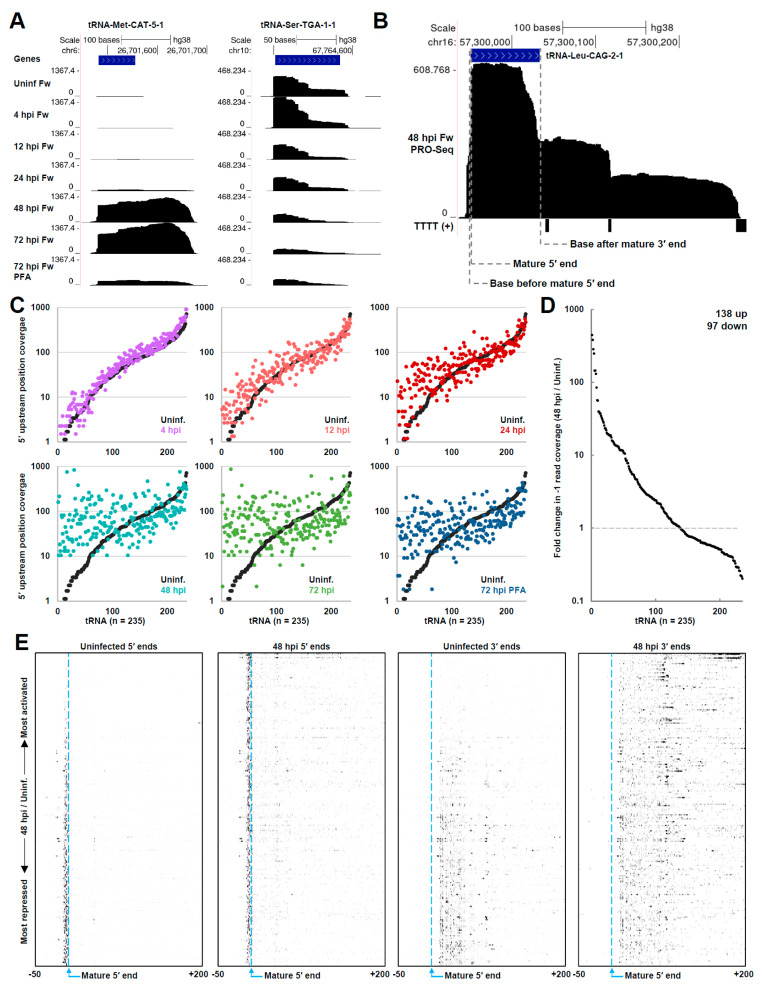
HCMV infection dramatically impacted Pol III transcription of tRNAs. (**A**) Genome browser snapshots of forward-strand (Fw) PRO-Seq data at tRNA genes that were significantly induced along the course of infection (left) repressed along the course of infection (right); (**B**) genome browser snapshot of forward-strand PRO-Seq data of the tRNA-Leu-CAG-2-1 gene at 48 hpi. Sites where read coverage was quantified for analysis are indicated. Matches to the motif TTTT on the nontemplate strand detected with the UCSC genome browser short match function are indicated; (**C**) plots of read coverage at the 5′ upstream position in uninfected HFFs and HFFs infected for 4, 12, 24, 48, or 72 h, and 72 h + PFA. In each plot, the read coverage for each tRNA in the uninfected HFFs is displayed with the data for the infection time point, and the data are sorted by lowest to highest read coverage in uninfected cells. Data are plotted on a log_10_ scale; (**D**) Graphs depicting the fold-change in read coverage at the 5′ upstream position (48 hpi/uninfected) for all 235 analyzed tRNA genes, sorted from most induced to most repressed. Fold change was plotted on a log_10_ scale; (**E**) heatmaps of PRO-Seq 5′ ends and 3′ ends at tRNA genes in uninfected HFFs and HFFs infected for 48 h. Data are displayed for a region spanning −50 to +200 relative to the mature 5′ end and are sorted from top to bottom by most induced at 48 h to most repressed according to the ratio of read coverage at the 5′ upstream position.

Dramatic changes in the transcription of most tRNAs were observed over the course of infection. For example, the transcription of tRNA-Met-CAT-5-1 was induced more than 400-fold at 48 hpi. Major increases in PRO-Seq signal were detected over the TSS and downstream of the mature tRNA 3′ end (Figure 5A, left). Interestingly, blocking HCMV replication with PFA was associated with only a partial induction of transcription, which suggests that accumulating levels of viral genomes and mRNA may drive tRNA induction. This result also suggests the involvement of a viral early-late or late factor in the effect on tRNA transcription, and is partly in keeping with a recent study, which showed that an HSV-1 mutant deficient for the viral DNA polymerase (UL30) did not induce tRNA expression to the same extent as the wild-type virus, nor did infected cells treated with DNA polymerase inhibitors [27]. By contrast, certain tRNAs, such as tRNA-Ser-TGA-1-1, were apparently repressed (Figure 5A, right). Interestingly, the PRO-Seq signal within tRNA-Ser-TGA-1-1 was characterized by sharp decreases that were not associated with termination signals and may represent Pol III pausing, which is further explored later. 

We noticed that some tRNAs transcripts had 5′ end signals coinciding with the mature 5′ end of the tRNA (Figure S3A). Many transcripts contained 3′ ends upstream of the mature 3′ end, indicating that they may be nascent transcripts. However, this could be due to transcription actually starting at the mature 5′ end, 5′ end processing occurring co-transcriptionally, or some amount of processed tRNA contamination. Because of this uncertainty, we developed a method to separate those transcripts from those that started upstream of the mature 5′ end (Figure 5B). Using the PRO-Seq signal for the tRNA-Leu-CAG-2-1 gene as an example, we quantified the number of reads at the mature 5′ end seen in the annotation, the number of reads crossing the preceding base position from transcription that started upstream of the mature 5′ end (termed the 5′ upstream position), and the number of reads crossing the base following the mature 3′ end (termed the 3′ downstream position) (Figure 5B, Supplementary Data File, tRNA Analyses). The short segment of the pre-tRNA between the TSSs and the mature 5′ end is referred to as the leader sequence and is removed during 5′ end processing. It was noted for tRNA-Leu-CAG-2-1 that PRO-Seq fragments beginning at the mature 5′ end were associated with 3′ ends within the tRNA gene body or close to the mature 3′ end, whereas fragments containing the leader sequence were primarily associated with 3′ ends downstream of the mature 3′ end, reflecting Pol III elongation (Figure S3B). Pol III terminates while synthesizing polyuridine tracts, which destabilize the elongation complex [73]. Accordingly, sharp decreases in engaged Pol III were detected precisely in line with poly-T stretches on the nontemplate strand (Figure 5B). For subsequent analyses, we used tRNA genes that had a ratio of read coverage at the base preceding the mature 5′ end over the reads starting at the mature 5′ end of 0.8 or greater (269/429, or 63% of annotated tRNAs). A minimum read cutoff for the analysis was also imposed, resulting in a final list of 235 tRNAs. That the majority of transcribed tRNAs contained upstream leader sequences and many, by observation, exhibited termination over poly-T stretches verified that PRO-Seq captured nascent Pol III transcripts. 

To view the global effects of HCMV infection on tRNA transcription, scatterplots of read coverage at the 5′ upstream position, representing initiation, were generated comparing the uninfected control to each infection time point (Figure 5C). The data were sorted by an increasing read coverage in the uninfected control. At early times postinfection (4 hpi), differences in tRNA transcription were subtle, but were notable at 12 hpi and marked by 24 hpi. At 48 and 72 hpi, the global profile of tRNA transcription was remarkably transformed. The blockage of HCMV replication with PFA partially attenuated global effects on tRNA transcription. Interestingly, tRNAs that were virtually silent in uninfected cells were activated, many more than 100-fold, while repressed tRNAs tended to correlate with those that were already highly transcribed in uninfected cells. Of the 235 analyzed tRNAs, 138 (59%) exhibited an increase in transcription, while 97 (41%) exhibited a decrease (Figure 5D). Interestingly, the induced population of tRNAs was modestly enriched for a set containing AT-rich anticodons (45% GC, 55% AT) versus those that were repressed (50% GC, 50% GC). The effects on tRNA transcription were also reproduced in samples treated with Flavo, which indicated that the dramatically altered occupancy of Pol III was not affected by the loss of Pol II productive elongation for 1h (Figure S3C). Coverage at the 5′ upstream position correlated poorly between the samples uninfected at 48 hpi (r = 0.18), as expected, but each of the mock infected or time-matched samples −/+ Flavo correlated well (r = 0.78 to 0.97). Similar effects on tRNA transcription were also observed by plotting the coverage over the base downstream of the annotated 3′ end in uninfected and infected samples (Figure S3D).

To view the effects on tRNA transcription on a gene-by-gene basis, heatmaps of tRNA 5′ and 3′ ends were generated spanning a region of −50 to +200 bp relative to the mature 5′ ends (Figure 5E). The genes were sorted by most induced at 48 hpi to most repressed, top to bottom. The position of the mature 5′ end was indicated. Notably, for virtually all tRNAs, PRO-Seq 5′ ends were located upstream of the mature 5′ end, indicating that the data represented true Pol III initiation. The apparently inverse relationship between tRNA genes that were active in uninfected cells and those that were most induced at 48 hpi was highly evident. With infection, PRO-Seq 3′ ends extended well past the mature 3′ end at most tRNA genes, which are on average 74 nt downstream of the mature 5′ end. Interestingly, a recurrent pattern of 3′ ends in the heatmap for the uninfected sample was evident, and this pattern was diminished at 48 hpi, reflected instead by a shift in 3′ ends downstream. Barring the possibility that these apparent pauses in Pol III transcription arose from an undiscovered mapping artifact linked possibly to tRNA gene duplication, it seems that infection could alter tRNA transcription, in part, through the stimulation of Pol III elongation. However, a more extensive investigation would be needed to test this hypothesis.

### 3.6. HCMV Infection Alters the Chromatin State of Pol III-Transcribed Genes

**Figure 6:**
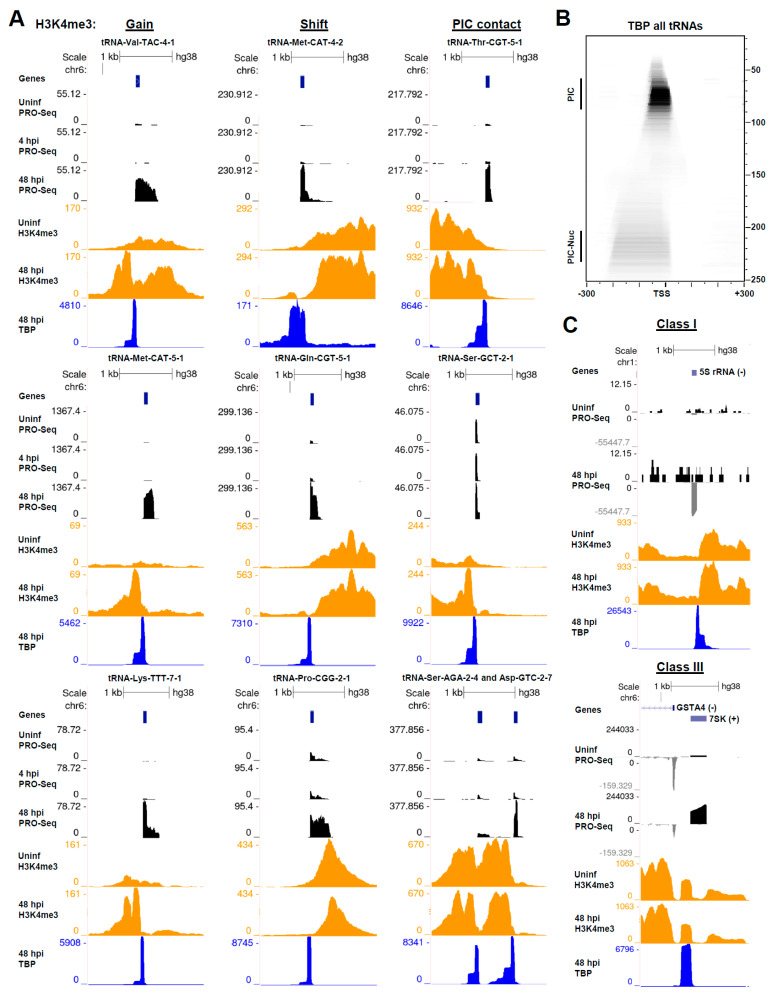
HCMV alters the chromatin state at induced tRNA genes. (**A**) Genome browser snapshots of forward strand PRO-Seq data in uninfected HFFs and HFFs infected for 4 or 48 h, DFF-ChIP for H3K4me3 in uninfected HFFs and HFFs 48 hpi, and DFF-ChIP for TBP in HFFs 48 hpi, at tRNA genes that represented three distinct changes in local chromatin observed in association with HCMV infection (gain in H3K4me3, shift in H3K4me3 nucleosomes, and increased contact between the −1 nucleosome and the Pol III PIC). Three examples of each effect are shown; (**B**) FragMap of TBP DFF-ChIP data tRNA genes centered on the tRNA max TSS. The location of the Pol III PIC with respect to the TSS and association between the Pol III PIC and the upstream −1 nucleosome are visible; (**C**) genome browser snapshots of PRO-Seq data and DFF-ChIP data for H3K4me3 and TBP revealing an induction of the Class I 5S rRNA gene and Class III 7SK gene by HCMV infection, and features of TBP-containing PICs at these Pol III promoters.

Next, we investigated whether changes in Pol III transcription induced by infection were associated with changes in the chromatin structure around Pol III-transcribed genes, once more drawing upon our recently published DFF-ChIP data for H3K4me3 and TBP [18]. Similar to Pol II promoters, tRNA genes are couched within accessible regions of the genome [74]. H3K4me3 was also detected around Pol III promoters, though the abundance of this modification was often considerably less than that observed at Pol II promoters, and there was no consistent pattern of H3K4me3 flanking tRNA genes. In some cases, neighboring Pol II transcription impacts tRNA expression [9] and is likely to influence the epigenetic environment of tRNA genes. Three distinct effects on H3K4me3 were observed in association with tRNA induction by infection. Some tRNAs, such as tRNA-Val-TAC-4-1, tRNA-Met-CAT-5-1, and tRNA-Lys-TTT-7-1, which were nearly silent in uninfected cells, exhibited a marked gain in H3K4me3 in association with their induction (Figure 6A, left). At induced tRNAs, where H3K4me3-modified nucleosomes were detected downstream of the tRNA gene, such as tRNA-Met-CAT-4-2, tRNA-Gln-CGT-5-1, and tRNA-Pro-CGG-2-1, a downstream shift in the proximal H3K4me3 nucleosomes was often observed, which may be related to increased levels of Pol III elongation downstream of the tRNA gene (Figure 6A, middle). DFF-ChIP for TBP revealed prominent signals corresponding to Pol III PICs that, interestingly, were almost always associated with an upstream extension of approximately 150 bp that appeared to reflect association of the PIC with the -1 nucleosome (Figure 6). In yeast, the -1 nucleosome is more strongly positioned at tRNA genes than the downstream +1 nucleosome [74]. This is at contrast with Pol II promoters, where the +1 nucleosome was more strongly positioned, and interactions between the Pol II PIC and +1 nucleosome were detectable [18]. In view of this finding, at tRNA genes where H3K4me3 nucleosomes were detected primarily upstream, such as tRNA-Thr-CGT-5-1, tRNA-Ser-GCT-2-1, tRNA-Ser-AGA-2-4, and tRNA-Asp-GTC-2-7, stronger positioning of the −1 nucleosome was detected, and the signal profiles of H3K4me3 data suggested an increased contact between the −1 nucleosome and the PIC −/+ infection (Figure 6A, right). A fragMap for TBP at all tRNA genes revealed the size and position of the Pol III PIC relative to the major TSS and clearly showed an association between this PIC and the upstream −1 nucleosome (Figure 6B). The significance of this interaction was unclear, but it is possible that maintaining an interaction between the Pol III PIC and −1 nucleosome could block Pol II PIC assembly and transcriptional interference. Pol III also transcribes the 5S rRNA and a variety of snRNAs, including 7SK, which contain Class I and Class III promoters, respectively. An investigation of our PRO-Seq and DFF-ChIP data suggested that Pol III transcription of these genes was also induced by infection. Relating to this, it was previously noted that the Epstein–Barr virus induces the expression of 5S rRNA and 7SL [31]. Our TBP DFF-ChIP data revealed a unique struc-ture to PICs at Class I and Class III genes as compared to Class II tRNA genes, pre-sumably reflecting the involvement of different general transcription factors in the as-sembly of the complex. A diagram that depicts the size of the protected region detected by TBP DFF-ChIP in relation to the TSS for each promoter subtype, in addition to Pol II and Pol I promoters, is provided in Figure S4.

## 4. Discussion

Here, we investigated the transcriptional effects of lytic HCMV infection in HFFs using PRO-Seq, which profiles nascent transcripts being generated by Pol I, II, and III. Our study provided surprising new insights, revealing, most significantly, that late HCMV infection was associated with a nearly global increase in the rate of the release of Pol II into productive elongation, that infection was seemingly linked to a change in Pol I transcription of the 45S rRNA at the level of elongation, and that, like HSV-1 and MHV68 [27,28], HCMV dramatically impacted tRNA expression at the level of transcription. These findings were connected to chromatin and epigenetic changes at affected genes, and together significantly extended our understanding of how HCMV manipulates the transcription of the host genome.

Our PRO-Seq data unexpectedly showed that that levels of Pol II productive elongation were substantially increased at late times postinfection (48, 72 h), while levels of initiation appeared to be largely unaffected. This observation is in alignment with the idea that HCMV does not shut off transcription of host genes as is thought to occur in the context of HSV-1, KSHV, and MHV68 infection. The trend was observed at most actively transcribed genes (Figure 1B and Figure 2A,B) and appeared to be dependent on viral genome replication, as treatment with PFA from the onset of a 72 h infection restored pause ratios to a prereplication level (24 h). Pol II pause release is achieved by the action of P-TEFb, which is recruited to pause regions by BRD4 and other transcriptional coactivators, and critically phosphorylates the pausing and productive elongation factor DSIF. The phosphorylation of DSIF leads to the dissociation of the pausing factor NELF, after which the elongation complex is engaged by productive elongation factors to facilitate elongation through the gene body [14,75]. In cells, a portion of P-TEFb is free and able to mediate pause release, while the remainder exists within an inhibitory 7SK snRNP. P-TEFb is directly controlled by certain viruses, such as HIV, to drive productive viral gene expression [76], and P-TEFb release from the 7SK snRNP may be an important effector of HSV-1 reactivation from latency [77]. P-TEFb is generally required for productive transcription at the HCMV genome [12], reflective of its general role in Pol II transcription. At late times, following the onset of the HCMV genome replication, the transcription of the HCMV genome accounted for ~10–25% of the transcription in the cell (Supplementary Data File, PRO-Seq Stats), and, thus, a substantial fraction of free cellular P-TEFb must be needed to drive productive elongation on the HCMV genome. A previous study indicated that total P-TEFb levels are increased in HCMV-infected cells and that Cdk9 localization is altered during early and late times postinfection [78]. An increase in P-TEFb levels may explain our observed effects on pause release. Whether HCMV impacts the fraction of P-TEFb present in the snRNP or the total amount of P-TEFb is unknown and should be explored. In addition to uncovering this previously unreported broad effect of HCMV infection on pausing and productive elongation, our PRO-Seq data identified subsets of genes, consisting of many ISGs that were induced with differential kinetics in response to infection, and using DFF-ChIP for H3K4me3, we also showed that genes repressed by HCMV infection exhibited NFR closure.

Our PRO-Seq data unexpectedly suggested that Pol I transcription of the 45S rRNA is impacted by HCMV infection. Relatively few studies have investigated Pol I initiation and elongation in cells using next-generation sequencing approaches, which is in part related to limitations of existing annotations of rDNA repeats. Our data suggests that there is an enrichment of elongating Pol I at the 5′ end of the 45S gene, which is reminiscent of Pol II pausing, and that late HCMV infection was associated with a decrease in this 5′ pileup and increased levels of Pol I elongation downstream. This effect also appeared to be dependent on viral replication, increased levels of viral mRNA, and/or a viral factor, as treatment with PFA led to its partial reversal. These data suggest that HCMV may drive productive rRNA transcription and perhaps downstream ribosome biogenesis to facilitate robust translation of viral mRNAs in a competitive environment where host mRNAs remain abundant. Additionally, our DFF-ChIP data for TBP sharply demarcated the boundaries of Pol I PICs in the 45S promoter region, and unexpectedly revealed PICs downstream of 45S that drive low levels of transcription initiation, are intimately associated with TTF1 termination sites, and could be involved in insulating tandem rDNA repeats from upstream Pol I transcription.

Finally, our PRO-Seq data revealed that HCMV dramatically induced the transcription of tRNAs. Our use of PRO-Seq to address effects on tRNA transcription is novel. Standard RNA-Seq approaches fail to accurately quantify the abundance of mature tRNAs due to extensive base modifications and secondary structure, which are both refractory to reverse transcription. Resultantly, methods that partially overcome these obstacles and enable a quantitative assessment of the levels of both mature tRNAs and pre-tRNAs have been developed [69,70,71,79,80]. PRO-Seq differs in that Pol III nascent transcripts are captured and sequenced. As tRNAs are post-transcriptionally processed, nascent Pol III transcripts lack base modifications. Also, the secondary structure within incompletely synthesized tRNAs may be minimal. Enrichment for nascent Pol III transcripts by PRO-Seq was shown through abundant evidence of unprocessed tRNA leader sequences, Pol III elongation downstream of the mature tRNA 3′ end, and termination over sites of poly-U incorporation. An advantage to measuring nascent transcripts is that mature tRNA half-lives are long, on the order of days, and, thus, small changes in the level of mature tRNAs could belie a major change at the level of transcription. In addition, effects on nascent transcription measured by PRO-Seq are likely to directly correlate with downstream changes in the levels of mature tRNA. We found that HCMV infection primarily resulted in an upregulation of tRNA transcription. Many tRNAs were induced more than 10-fold, and it was noted that induced tRNAs were modestly biased towards those containing AT-rich anticodons compared to repressed tRNAs. Interestingly, viral genome replication appeared to be important for the full induction of tRNA transcription, as treatment with PFA shifted the induction of tRNAs to prereplication levels observed at 24 h. This result suggests that tRNA induction may be linked to an increasing abundance of viral mRNAs, a result that is mirrored by a recent study of HSV-1 [27], and suggests the involvement of a viral factor in Pol III stimulation. Interestingly, and apparently unique to HCMV, the most induced tRNAs tended to be transcribed at low levels in uninfected cells, while repressed tRNAs tended to be very actively transcribed in uninfected cells. Thus, it appears that viruses from all major human herpesvirus subfamilies impact tRNA expression, possibly to the benefit of productive infection. 

Interestingly, recent reports have suggested that HSV-1 and MHV68 generally lead to a greater increase in levels of pre-tRNAs than mature tRNAs [27,28]. It is possible that immature tRNAs may give rise to tRNA-derived fragments, which have recently emerged as important regulators of gene expression at the post-transcriptional level [81]. Whether tRNA transcripts that are induced by HCMV infection undergo complete maturation remains to be discerned. Regarding a mechanism for tRNA induction by HCMV, one possibility relates to the induction of Pol III initiation machinery, which has been reported to occur in the context of EBV infection [31]. Another interesting possibility relates to the function of Maf1, a Pol III transcriptional repressor that blocks the function of TFIIIB in Pol III PIC assembly [82,83]. Maf1 is rendered inactive by phosphorylation, which is thought to be mediated, at least in part, by the mTOR kinase [84,85]. The mTOR complex is a major player in the regulation of cellular metabolism and translation, and viral infection often triggers a cellular stress response that inactivates mTOR and, consequently, leads to a global downregulation of mRNA translation [86]. However, the HCMV UL38 protein, which is expressed with early kinetics, is thought to subvert this response by binding to and inhibiting TSC2, which with TSC1 indirectly represses mTOR, thereby promoting mTOR function [87,88]. Notably, some tRNAs began to be induced at 4 and 12 hpi, which was possibly consistent with early UL38 function, and UL38 protein levels apparently increase between 24 and 48 hpi [88]. This action may not only enable the translation of viral mRNAs, but could also lead to Maf1 inactivation by mTOR-mediated phosphorylation, with downstream consequences for tRNA transcription. Relating to this, HSV-1 is also a known mediate constitutive in mTOR activation, even in the context of nutrient deprivation, using similar mechanisms that impinge on upstream mTOR effectors [89,90]. However, possibly at contrast with these ideas, it was demonstrated that MHV68 infection induced tRNA upregulation in wild-type and Maf1-deficient cells [28]. 

Finally, our DFF-ChIP data provided several new insights regarding the chromatin status of tRNA genes and how it is impacted by infection. We observed that some induced tRNA genes exhibited gains in H3K4me3 at proximal nucleosomes, while tRNAs that were associated with H3K4me3 nucleosomes downstream exhibited a shift in the downstream nucleosomes to a more distal position in association with tRNA induction. The latter may be the result of increased levels of Pol III elongation downstream of the tRNA. Finally, our DFF-ChIP data resolved a striking connection between Pol III PICs and the upstream −1 nucleosome. At induced tRNAs, the profile of H3K4me3 signal appeared to suggest stronger positioning of the −1 nucleosome and increased contact with the Pol III PIC. A caveat to these interpretations is that H3K4me3 is likely substoichiometric with respect to nucleosomes flanking tRNA genes. As such, future studies should explore changes in the tRNA chromatin structure using approaches that are agnostic to the epigenetic status. Overall, our results positioned PRO-Seq and DFF-ChIP as useful tools for investigating the dynamics of tRNA transcription and chromatin.

## 5. Conclusions

In this study, we showed that HCMV infection elicited major changes in transcription by Pol I, II, and III using PRO-Seq. Our investigation of Pol II transcription unexpectedly revealed a substantial increase in the rates of Pol II pause release at late times postinfection that was reflected at most genes. This is in contrast with the effects of alpha and gammaherpesviruses, which are thought to shut off host Pol II transcription. We showed that Pol I transcription was characterized by an apparent block to elongation at the 5′ end of the 45S gene that diminished during late infection, where increased levels of elongating Pol I were detected downstream. Finally, we reported on massive changes in Pol III transcription of tRNA genes, similar to recent reports on HSV-1 and MHV68 [27,28]. Hundreds of tRNAs were differentially transcribed during infection, and many were induced massively, up to nearly 500-fold. This effect largely required HCMV genome replication, indicating that tRNA induction may be coupled to increasing levels of viral mRNA or involve viral factors that remain to be identified.

## Data Availability

The raw and processed PRO-Seq and DFF-ChIP data analyzed in this study have been published in our previous study, and are available at NCBI GEO GSE185763.

## Supplementary Materials

The following are available online at https://www.mdpi.com/article/10.3390/v14040779/s1, Supplementary Figure S1: Motif enrichment analysis in clustered regions, Figure S2: Reproducibility of Pol I transcription profile, Figure S3: Reproducibility of Pol III findings, Figure S4: Schematic depicting the regions protected by DFF-ChIP for TBP at Pol I, Pol II, and Pol III promoters, Supplementary Data File.
