# Supplementary material for: Human Cytomegalovirus Infection Elicits Global Changes in Host Transcription by RNA Polymerases I, II, and III
Source: Viruses. 2022 Apr 9;14(4):779. doi: 10.3390/v14040779 (PMC9026722; doi:10.3390/v14040779)
Supplement: Supplementary file 1 [file viruses-14-00779-s001.zip › Supplementary Figures.pdf]

Supplementary Figures

Impact of HCMV infection on host transcription by RNA Polymerases I, II, and III

Christopher B. Ball[1], Mrutunjaya Parida[1], Ming Li[2], Benjamin M. Spector[1], Gustavo S. Suarez[1], Jeffery L. Meier[2], and David H. Price[1*]

[1] Department of Biochemistry and Molecular Biology, University of Iowa, Iowa City, IA, USA
[2] Departments of Internal Medicine and Epidemiology, University of Iowa and Iowa City Veterans Affairs Health Care System, Iowa City, IA, USA
* Correspondence: david-price@uiowa.edu

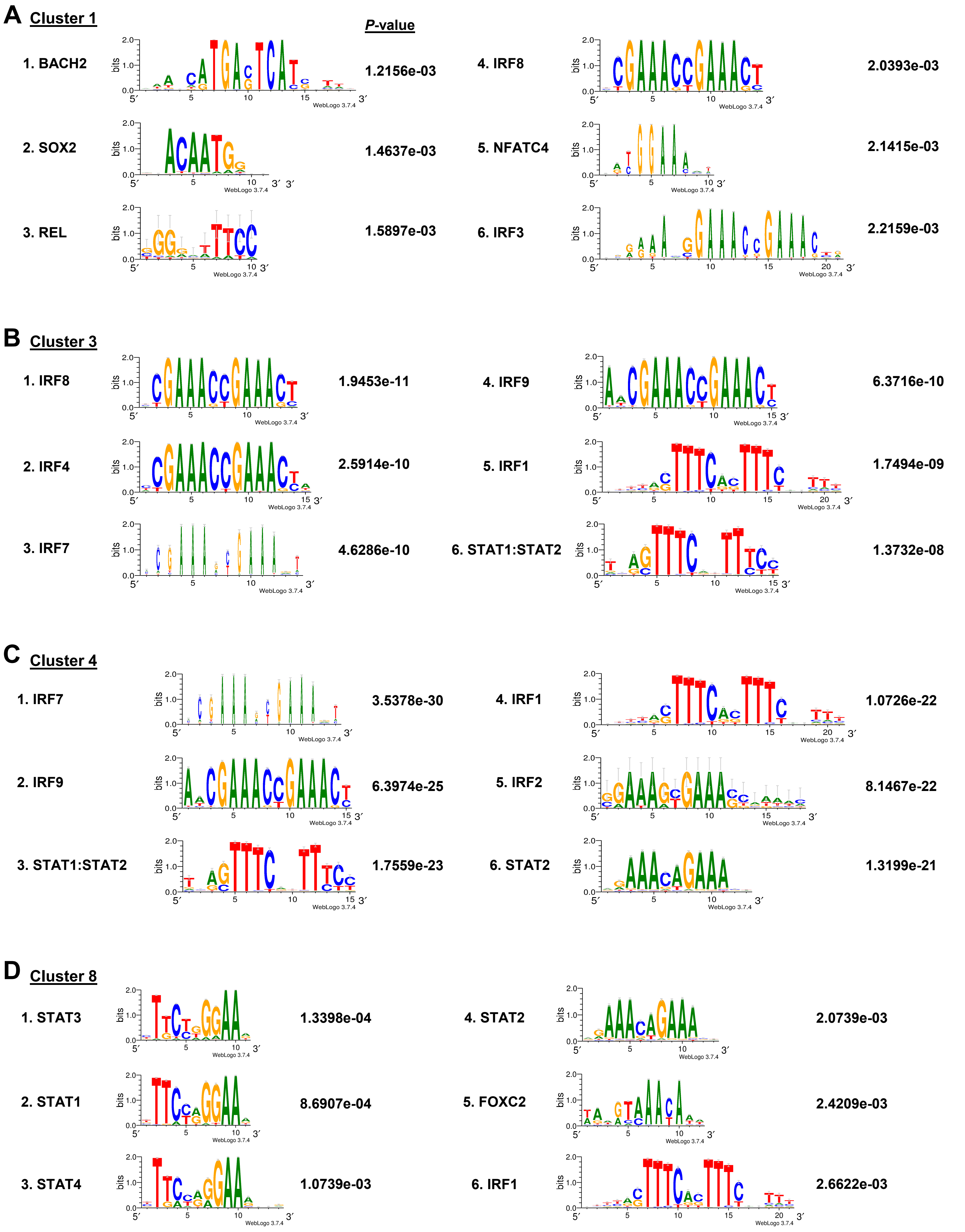
A
Cluster 1
P-value
1. BACH2
1.2156e-03
2. SOX2
1.4637e-03
3. REL
1.5897e-03
4. IRF8
2.0393e-03
5. NFATC4
2.1415e-03
6. IRF3
2.2159e-03
B
Cluster 3
1. IRF8
1.9453e-11
2. IRF4
2.5914e-10
3. IRF7
4.6286e-10
4. IRF9
6.3716e-10
5. IRF1
1.7494e-09
6. STAT1:STAT2
1.3732e-08
C
Cluster 4
1. IRF7
3.5378e-30
2. IRF9
6.3974e-25
3. STAT1:STAT2
1.7559e-23
4. IRF1
1.0726e-22
5. IRF2
8.1467e-22
6. STAT2
1.3199e-21
D
Cluster 8
1. STAT3
1.3398e-04
2. STAT1
8.6907e-04
3. STAT4
1.0739e-03
4. STAT2
2.0739e-03
5. FOXC2
2.4209e-03
6. IRF1
2.6622e-03
bits
WebLogo 3.7.4

Figure S1. Related to Figure 1. (A-D) Top 6 transcription factor motifs identified from motif enrichment analysis performed with promoter regions (150 bp pause region -200 bp and + 50 bp) for genes in clusters 1, 3, 4, and 8, respectively. The JASPAR motif for each transcription factor is shown, and to the right the P-value of enrichment (Fisher's exact test).

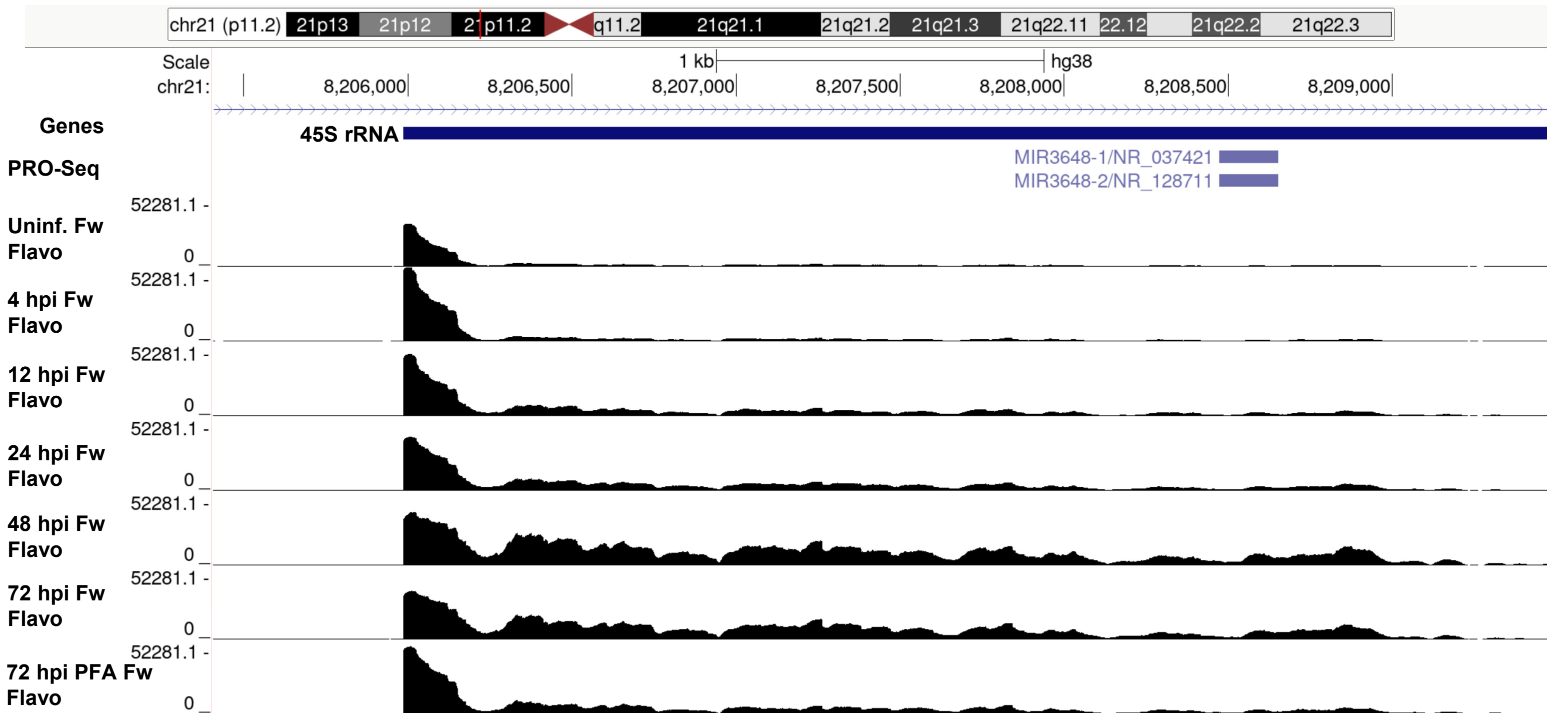

chr21 (p11.2)
21p13
21p12
21p11.2
q11.2
21q21.1
21q21.2
21q21.3
21q22.11
22.12
21q22.2
21q22.3
Scale
1 kb
hg38
chr21:
8,206,000
8,206,500
8,207,000
8,207,500
8,208,000
8,208,500
8,209,000
Genes
45S rRNA
MIR3648-1/NR_037421
MIR3648-2/NR_128711
PRO-Seq
Uninf. Fw Flavo
4 hpi Fw Flavo
12 hpi Fw Flavo
24 hpi Fw Flavo
48 hpi Fw Flavo
72 hpi Fw Flavo
72 hpi PFA Fw Flavo
52281.1
0

Figure S2. Related to Figure 4. Genome browser snapshot displaying Flavo PRO-Seq data along the infection time course at the 45S promoter region and upstream portion of the 45S rRNA that is not retained as a mature transcript.

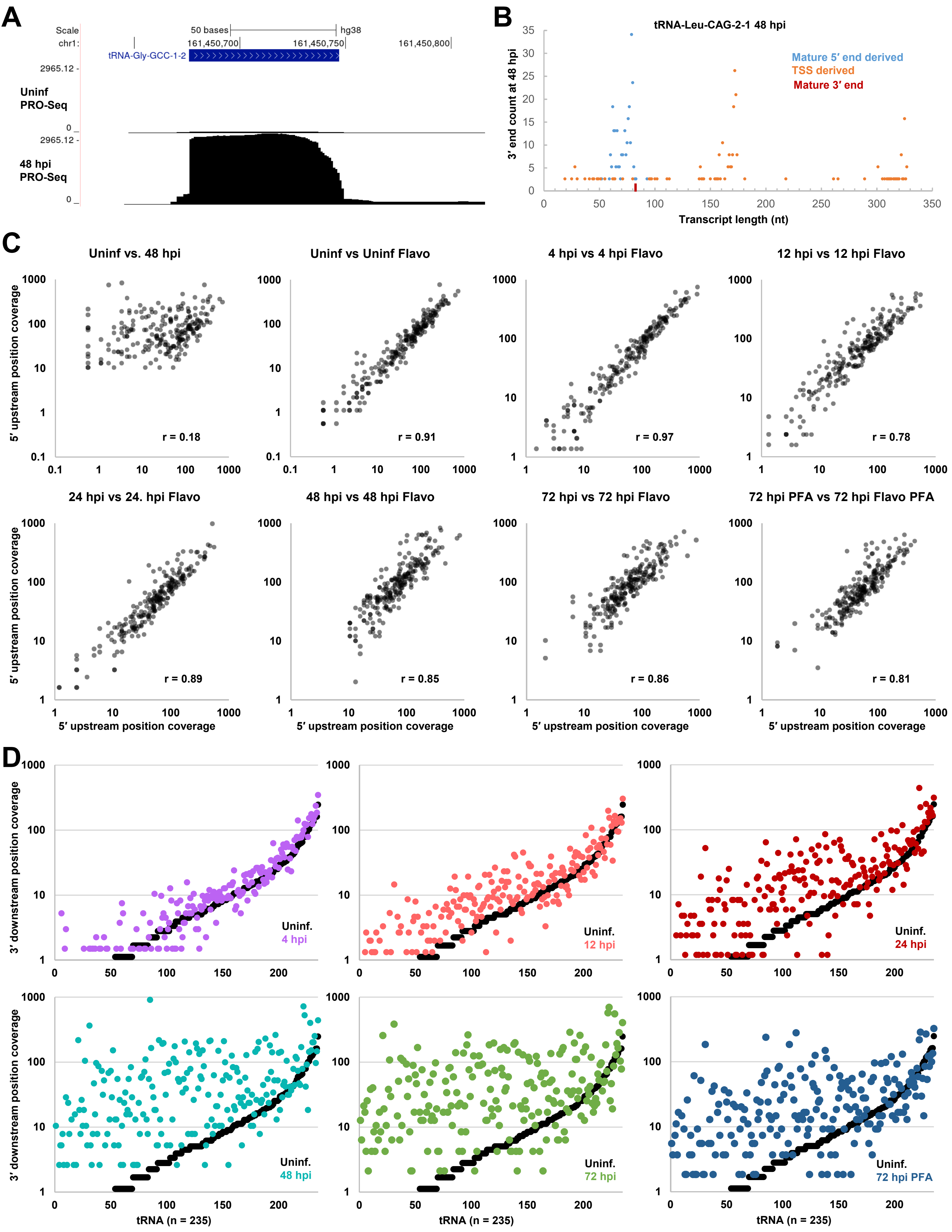

A
Scale
50 bases
hg38
chr1:
161,450,700
161,450,750
161,450,800
tRNA-Gly-GCC-1-2
2965.12 -
Uninf
PRO-Seq
0 _
2965.12 -
48 hpi
PRO-Seq
0 _
B
tRNA-Leu-CAG-2-1 48 hpi
Mature 5′ end derived
TSS derived
Mature 3′ end
3′ end count at 48 hpi
Transcript length (nt)
C
Uninf vs. 48 hpi
r = 0.18
Uninf vs Uninf Flavo
r = 0.91
4 hpi vs 4 hpi Flavo
r = 0.97
12 hpi vs 12 hpi Flavo
r = 0.78
24 hpi vs 24. hpi Flavo
r = 0.89
48 hpi vs 48 hpi Flavo
r = 0.85
72 hpi vs 72 hpi Flavo
r = 0.86
72 hpi PFA vs 72 hpi Flavo PFA
r = 0.81
5′ upstream position coverage
5′ upstream position coverage
D
3′ downstream position coverage
Uninf.
4 hpi
Uninf.
12 hpi
Uninf.
24 hpi
Uninf.
48 hpi
Uninf.
72 hpi
Uninf.
72 hpi PFA
tRNA (n = 235)

Figure S3. Related to Figure 5. (A) UCSC Genome browser snapshot of forward-strand PRO-Seq data at the tRNA-GCC-1-2 gene, which shows an abundance of PRO-Seq 5′ ends at the mature tRNA 5′ end and associated 3′ ends within the tRNA gene body. (B) Plot of length and density of 3′ ends associated with either the mature 5′ end (blue) or all upstream TSSs (orange). (C) Correlation analysis of read coverage at the 5′ upstream position for the uninfected and 48 hpi (DMSO) datasets, the mock infected-datasets -/+ Flavo, and time-matched infection points -/+ Flavo. The r value represents the Pearson's correlation coefficient. (D) Plots of read coverage at the base downstream 3′ position in uninfected HFF and HFF infected for 4, 12, 24, 48, or 72 h, and 72 h + PFA. In each plot, the read coverage for each tRNA in the uninfected HFF is displayed with the data for the infection time point, and the data are sorted by lowest to highest read coverage in the uninfected cells. Data are plotted on a $\log_{10}$ scale.

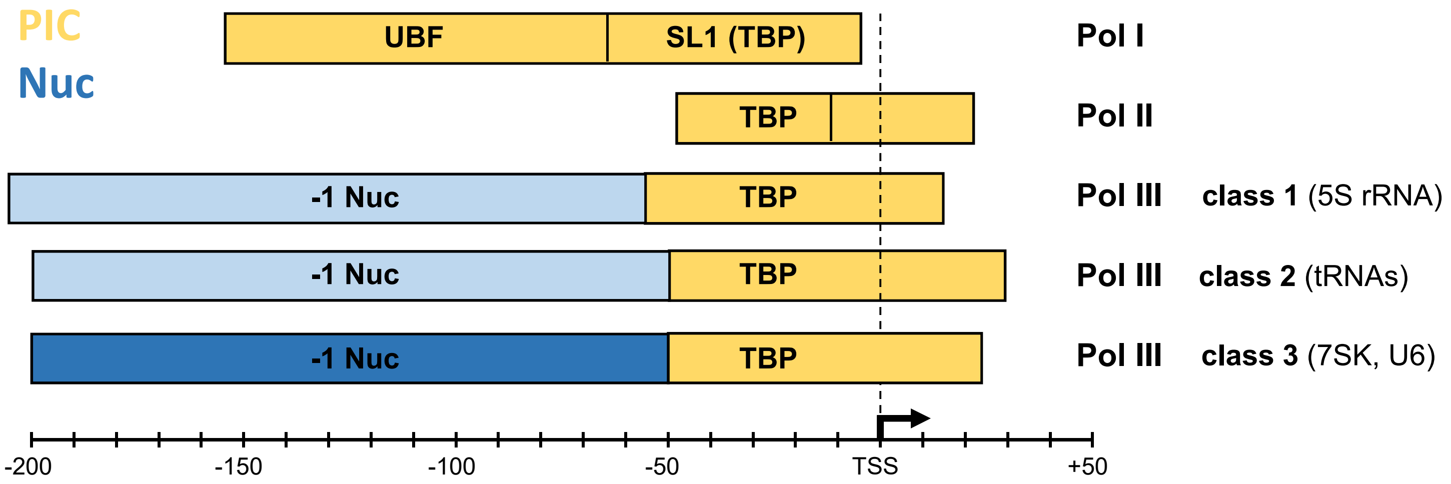
PIC
Nuc
UBF
SL1 (TBP)
Pol I
TBP
Pol II
-1 Nuc
TBP
Pol III class 1 (5S rRNA)
-1 Nuc
TBP
Pol III class 2 (tRNAs)
-1 Nuc
TBP
Pol III class 3 (7SK, U6)
-200
-150
-100
-50
TSS
+50

Figure S4. Related to Figure 6. Schematic depicting the regions protected by DFF-ChIP for TBP at Pol I, Pol II, and Pol III promoters (including Class I, II, and III sub-types). At Pol III promoters, the approximate position of the upstream -1 nucleosome associated with the PIC is indicated. The occupancy of the nucleosome relative to the PIC indicated by shading. At Class I and II Pol III promoters, an upstream nucleosome is detected in association with a fraction of Pol III PICs, whereas at Class III promoters, the PIC and upstream nucleosome exhibit approximately 100% co-occupancy.
